# Understanding barriers to immunization among women with zero-dose or under-immunized children in an urban area in Cameroon

**DOI:** 10.3389/fpubh.2026.1783886

**Published:** 2026-04-30

**Authors:** Ehouzou Mandeng, Mbono Bekoto Ritha, Adalbert Tchetchia, Mbengue Jeanne Liliane, Maffo Fonkwo Vanessa, Tchouaffi Awah Kum, Edzoa Essomba Brice, Ngo Um Sap Suzanne

**Affiliations:** 1 Faculty of Medicine and Pharmaceutical Sciences, University of Ebolowa, Ebolowa, Cameroon; 2 Faculty of Medicine and Pharmaceutical Sciences, University of Douala, Douala, Cameroon; 3 Framework for African Improvement in Research, Yaoundé, Cameroon; 4 Expanded Program on Immunization, Yaoundé, Cameroon; 5 Nylon Health District, Douala, Cameroon; 6 Department of Fundamental Education, Faculty of Education, University of Yaoundé, Yaoundé, Cameroon

**Keywords:** Cameroon, children, immunization, vaccination barriers, zero-dose children

## Abstract

**Background:**

Zero-dose children represent a major public health challenge in low-income countries. In 2024, the Nkolndongo Health District in Yaoundé, Cameroon, recorded the highest number of zero-dose children in the country, according to the District Health Information Software 2 (DHIS2) data.

**Objective:**

This study aimed to identify and describe the main reasons for non-vaccination and the obstacles to vaccination among zero-dose and under-immunized children in the Ndolndongo Health District, Yaoundé.

**Methods:**

A cross-sectional study was conducted from December 2024 to July 2025. The study used a convergent parallel mixed-methods design across three purposively selected priority health areas of the Nkolndongo Health District in Yaoundé (Nkolo, Essomba, Mimboman 2). Under-immunized children were defined as those who had missed at least one scheduled vaccine dose at the time of the study. This study included 50 women with zero-dose or under-immunized children, identified by community health workers or leaders of women’s associations, through consecutive, non-exhaustive sampling. Vaccination status was verified using vaccination cards and health facility registrars. Data collection included both quantitative (questionnaires) and qualitative (focus groups and interviews). The qualitative analysis was conducted using NVivo and Atlas.ti, with thematic analysis and grounded theory principles, while the quantitative analysis was conducted using SPSS version 26.0.

**Results:**

Twenty-three percent (23%) of children were classified as zero-dose. Mothers were predominantly single (74%), had secondary education (70%), and lived in poverty (66% had less than 50,000 Central African CFA francs, FCFA, per month). The most frequent reasons for non-vaccination were forgetting appointments (23%) and previous experience of adverse events following immunization (19%). Qualitative analysis revealed multiple barriers, including vaccine safety concerns, negative impacts of the coronavirus disease 2019 (COVID-19) pandemic, social media misinformation, inadequate sensitization, emotional factors, sociocultural beliefs, health service dysfunction, and logistical constraints.

**Conclusion:**

Within this study population, zero-dose children require multifaceted interventions targeting misinformation, strengthening health systems, adapting services to mothers’ constraints, and engaging entire communities, including male partners and vulnerable populations.

## Introduction

Zero-dose children are those who have not received any routine vaccines, including Penta 1 at 6 weeks (1, 2). Under-immunized children are defined as those who have initiated but not completed the recommended vaccination schedule for their age (1, 2). In the existing epidemiological context, the increasing number of zero-dose children represents a major public health challenge in low-income countries. These children are predominantly found in marginalized communities, where the health systems are fragile and social inequalities are pronounced (3, 4).

According to the literature, mothers of zero-dose children or under-immunized children (ZD/UI) generally present a characteristic profile: they are young, single, have low educational attainment, and are unemployed (5, 6). They reside in socioeconomically disadvantaged environments with restricted access to prenatal care (7, 8). Furthermore, vaccine hesitancy, fueled by misinformation and religious considerations, constitutes an additional obstacle in meeting vaccination needs (9). In the specific context, Cameroon’s rapid urban growth has led to the emergence of precarious housing areas where access to vaccination services has not kept pace with demographic expansion (7, 8). An accurate description of this profile is important to address the ZD/UI issues, according to the Gavi 5.0 strategy (10).

In Cameroon, the Nkolndongo Health District (HD) is among those with the highest number of zero doses in 2024. This district, located in Yaounde and in an urban setting, was particularly relevant for examining barriers to immunization among women with ZD/UI children. This study, therefore, aimed to identify and describe the main reasons for non-vaccination and the obstacles to the identification and vaccination of zero-dose and under-immunized children in this urban setting.

## Methodology

This study conducted a cross-sectional study using a convergent parallel mixed-methods design. During this study, quantitative and qualitative data were collected concurrently, analyzed separately, and merged during interpretation to provide a comprehensive understanding of vaccination barriers. Data collection took place from December 2024 to July 2025.

### Study setting

The study site was the Nkolndongo HD, located in the 4th subdivision of Yaoundé. This district comprises 11 health areas distributed over 57 km^2^. Nkolndongo HD has 617,236 inhabitants; the majority of whom are under 20 years old. The health infrastructure includes 194 health facilities, of which 89 (45.8%) provide vaccination services. Among these services, only 11 cold chain equipment units are available, of which 5 are certified. Socially, 174 communities cohabitate in the district, of which approximately 20 (12%) face geographical access difficulties.

### Sampling and participant selection

Health area selection was conducted purposefully. Three priority health areas were selected: *Nkolo, Essomba, and Mimboman 2*. The selection criteria were based on low Penta 1 vaccination coverage, a high number of ZD/UV in DHIS2, the presence of precarious neighborhoods, geographical accessibility constraints, and expert opinion from the health district management team.

For this study, zero-dose children were defined as children under 5 years of age who had not received any routine vaccine dose, including Penta1. Under-immunized children were defined as those who had initiated but not completed the vaccination schedule for their age group. Children aged over 5 years were excluded because the national vaccination schedule targets children up to 59 months, and their immunization history was considered too distant to allow reliable or valid card verification.

Women with ZD/UI were identified by community health workers (CHWs) using a door-to-door approach and by women leaders of community-based organizations (CBOs) through word of mouth. Both CHWs and CBO leaders were trained by the research team to identify women with ZD/UI in their areas. The sampling was consecutive and non-exhaustive, meaning that all eligible women identified during the study period were approached until the end of the data collection phase; no formal sample size calculation was applied. For qualitative data, the study continued sampling until theoretical saturation was reached.

Vaccination status was verified using vaccination cards when available, supplemented by health facility registers. In cases where neither source was available, maternal recall was used and documented.

*Sample justification*: The sample size was not calculated *a priori* for inferential statistical purposes. Given the exploratory nature of this study and significant operational constraints, including a 44% reduction in the planned intervention period, only 50 participants were included consecutively. Quantitative findings should therefore be interpreted as descriptive and hypothesis-generating rather than statistically conclusive. For the qualitative strand, sampling continued until theoretical saturation was reached.

### Data collection

For qualitative data collection, the study conducted 5 focus group discussions (FGDs) and 17 individual interviews using a semi-structured interview guide. These tools were previously tested and validated. Quantitative data were collected through questionnaires and vaccination registries.

### Data analysis

The study performed both quantitative and qualitative analyses. For qualitative analysis, data were extracted verbatim and analyzed using NVivo and Atlas.ti.

Nvivo was used for initial coding and thematic organization of FGD data. Atlati.ti was used for conceptual network mapping of themes from individual interviews, allowing cross-validation across data sources. Thematic analysis was prioritized, complemented by grounded theory principles, allowing inductive emergence of themes. Coding was conducted independently by two analysts; discrepancies were resolved through discussion and consensus. Theoretical saturation was considered achieved when no new themes emerged across the final interviews and FGDs. Triangulation was performed by cross-referencing findings from focus groups, individual interviews, and quantitative data to strengthen the validity of emerging themes.

Quantitative analysis was performed using Statistical Package for the Social Sciences (SPSS) version 26.0. All quantitative results are presented only as descriptive statistics (absolute frequencies and percentages). Given the small sample size and the exploratory design, no inferential statistical tests were applied.

### Ethical considerations

This study involves human subjects. All procedures complied with the Declaration of Helsinki and applicable national regulations. Ethical approval was obtained from the Regional Ethics Committee for Human Health Research of the Centre Region (Reference number 002400/05-06-2025). Administrative authorization was also granted by the Regional Delegation of Public Health before data collection and field activities.

Written informed consent was systematically obtained from all participants after a detailed explanation of objectives, procedures, potential risks, and expected benefits. Participants were informed of their right to withdraw at any time without prejudice. Confidentiality was ensured by assigning anonymous identification codes to each participant and excluding all nominative information from databases. Collected data were securely stored on password-protected digital media, accessible only to research team members.

The study encountered significant gender-related access barriers: 18% of identified families were represented by male partners rather than the women themselves. These cases were systematically excluded from analysis. Two women interrupted the interview to seek their husbands’ prior authorization. This pattern is documented as a substantive finding reflecting structural constraints on women’s autonomous participations in health research in this context. These measures aimed to guarantee respect for participants’ autonomy, dignity, and privacy throughout the process.

### Results

### Sociodemographic characteristics of mothers ZD/UI

**Figure 1:**
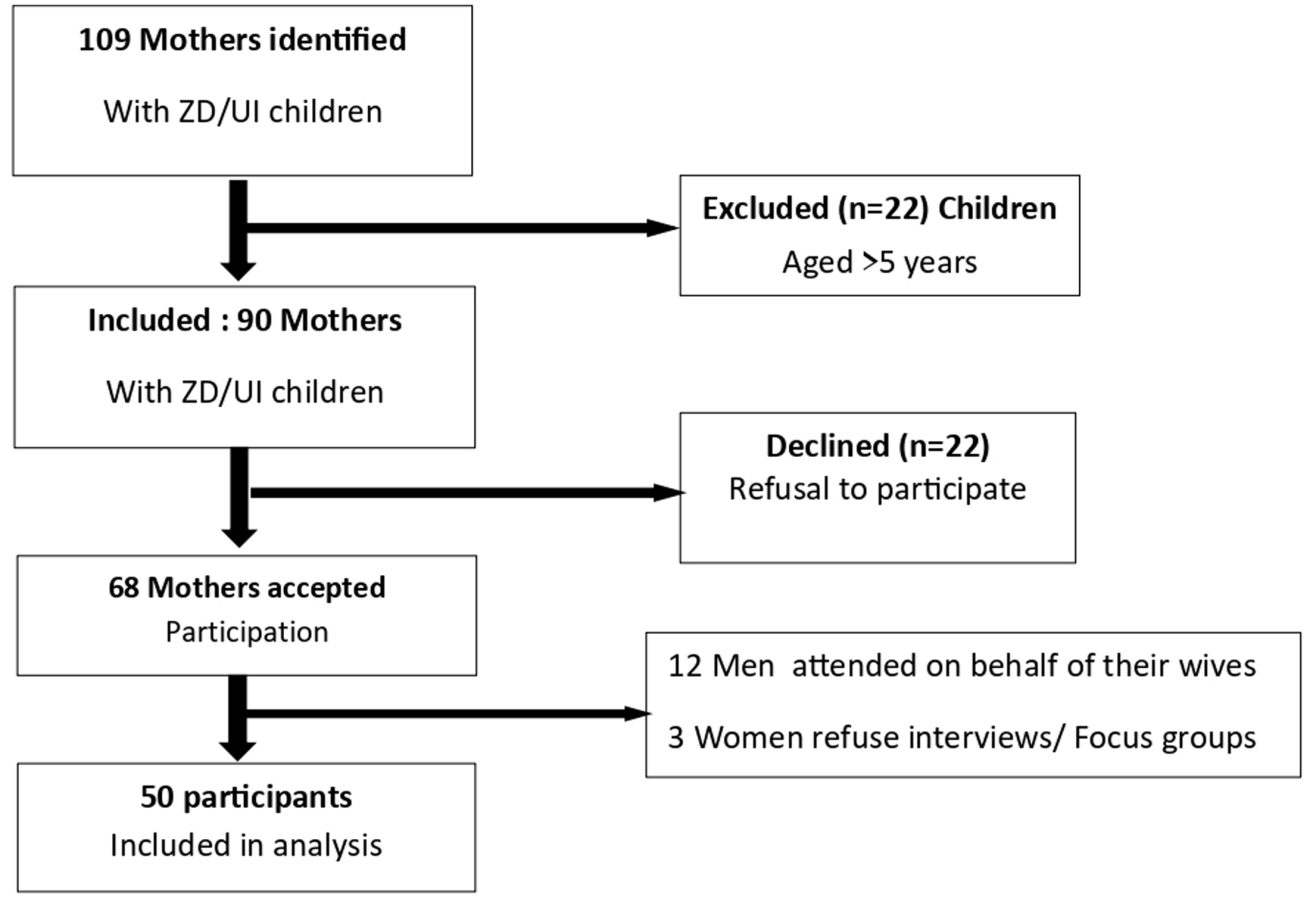
Participant selection and enrollment flowchart.

The study identified 109 mothers with ZD/UI and excluded 22 participants whose children were aged >5 years. Of the 90 eligible families, 68 accepted participation; of these, 12 men came on behalf of their wives, and 3 women refused to participate in interviews and focus groups (Figure 1). Half of the participants were aged 30–39 years, while only 2% were adolescents (Table 1). Three-quarters of mothers were single (74%). Regarding education level, 70% had reached the secondary level, 1 of 5 had reached the university level, and 4% had no schooling. More than one-third of mothers were unemployed (38%), and 28% worked in the informal sector. Monthly income was below 50,000 XAF (80 US dollars) for 70% of participants. A quarter of them had monthly incomes between 50,000 and 100,000 XAF (80–160 US dollars), and only 10% had incomes above 100,000 XAF (greater than 160 US dollars). Most mothers had two children or fewer (*n* = 28, 56%), 38% (*n* = 19) had 3 to 4 children, and large families (≥5 children) represented 6% (*n* = 3). Regarding vaccination status, 23% of the children were zero-dose, half had missed more than four vaccination contacts, and only 19% had missed only one contact (Table 1).

**Table 1:** Sociodemographic features of women ZD/UV.

Items	Frequency (*N*)	Percentage (%)
Age group		
<20	1	2
20–29	17	34
30–39	25	50
>40	7	14
Level of education		
None	2	4
Primary	3	6
Secondary	35	70
University	10	20
Marital status		
Single	37	74
Married or in couple	11	22
Widow	1	2
Divorced	1	2
Professional status		
Unemployed	19	38
Informal sector	14	28
Civil servant	4	8
Private sector	13	26
Declared family monthly income		
<50,000	32	64
50,000–100,000	13	26
>10,000	5	10
Number of children		
<2	28	56
<2–4	19	38
≥5	3	6

### Main causes of non-vaccination

#### Knowledge and perceptions

Participants recognized vaccination as an effective preventive method for childhood diseases. They knew the vaccination schedule and its recent modifications (catch-up, extension after 1 year). One participant stated, “*Vaccination protects the child against childhood diseases. It’s free and organized by the state.”*

**Figure 2:**
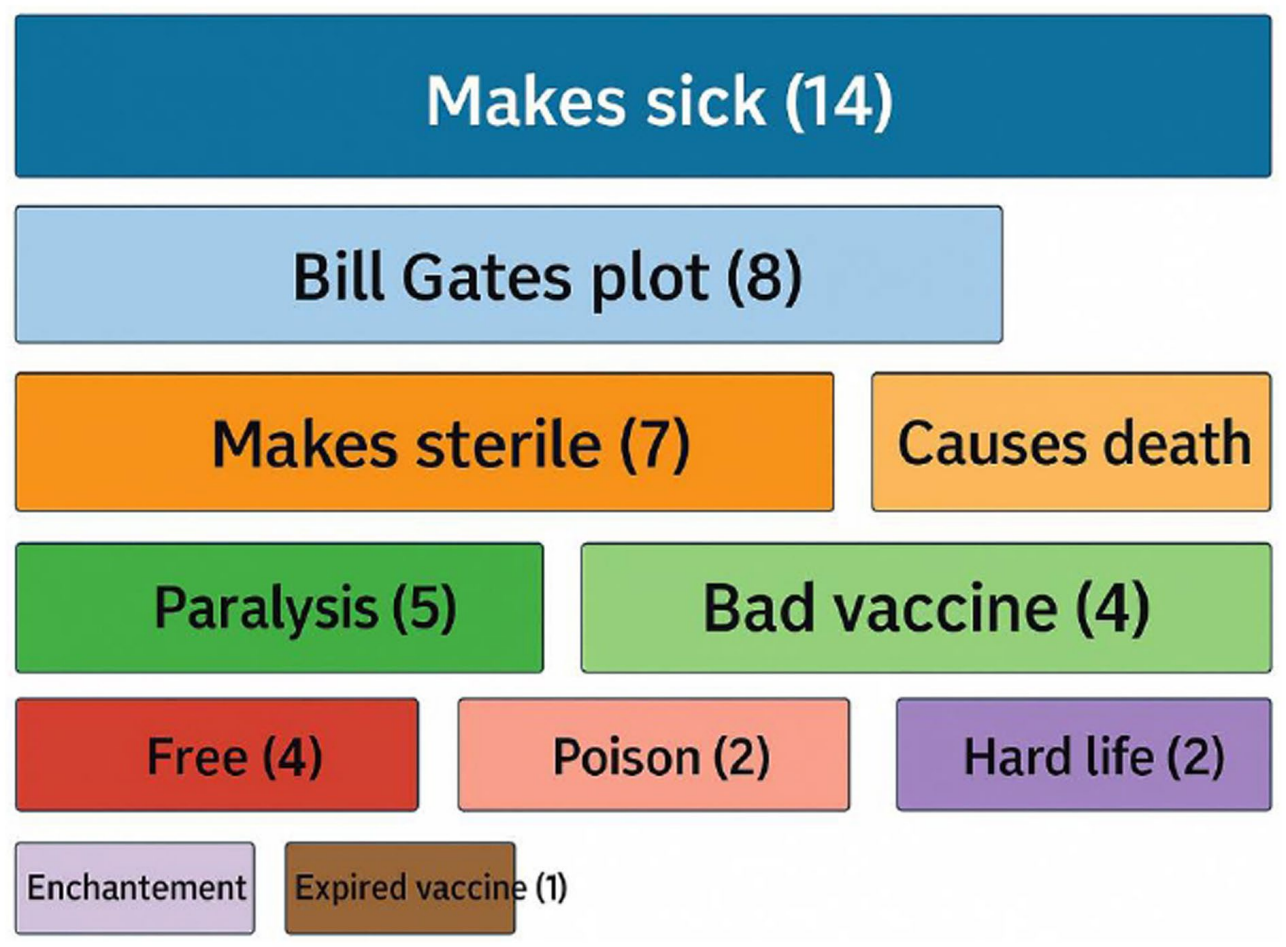
Main rumors on immunization and vaccines.

Despite this knowledge, rumors about vaccine safety circulated, generating mistrust. These were perceptions reported by participants; they are not supported by scientific evidence but constitute real barriers requiring targeted communication responses. The main reported beliefs were that vaccines make children sick (8/25%), cause paralysis (5/15.7%), and cause sterility (7/21.8%). Other participants mentioned expired or contaminated vaccines (4/12.5%) and conspiracy theories linked to “Bill Gates” (8/25%). “What’s scary is what happened in Kribi. The child was vaccinated and...” *[find another quote],* Figure 2.

### Reasons for non-vaccination

**Figure 3:**
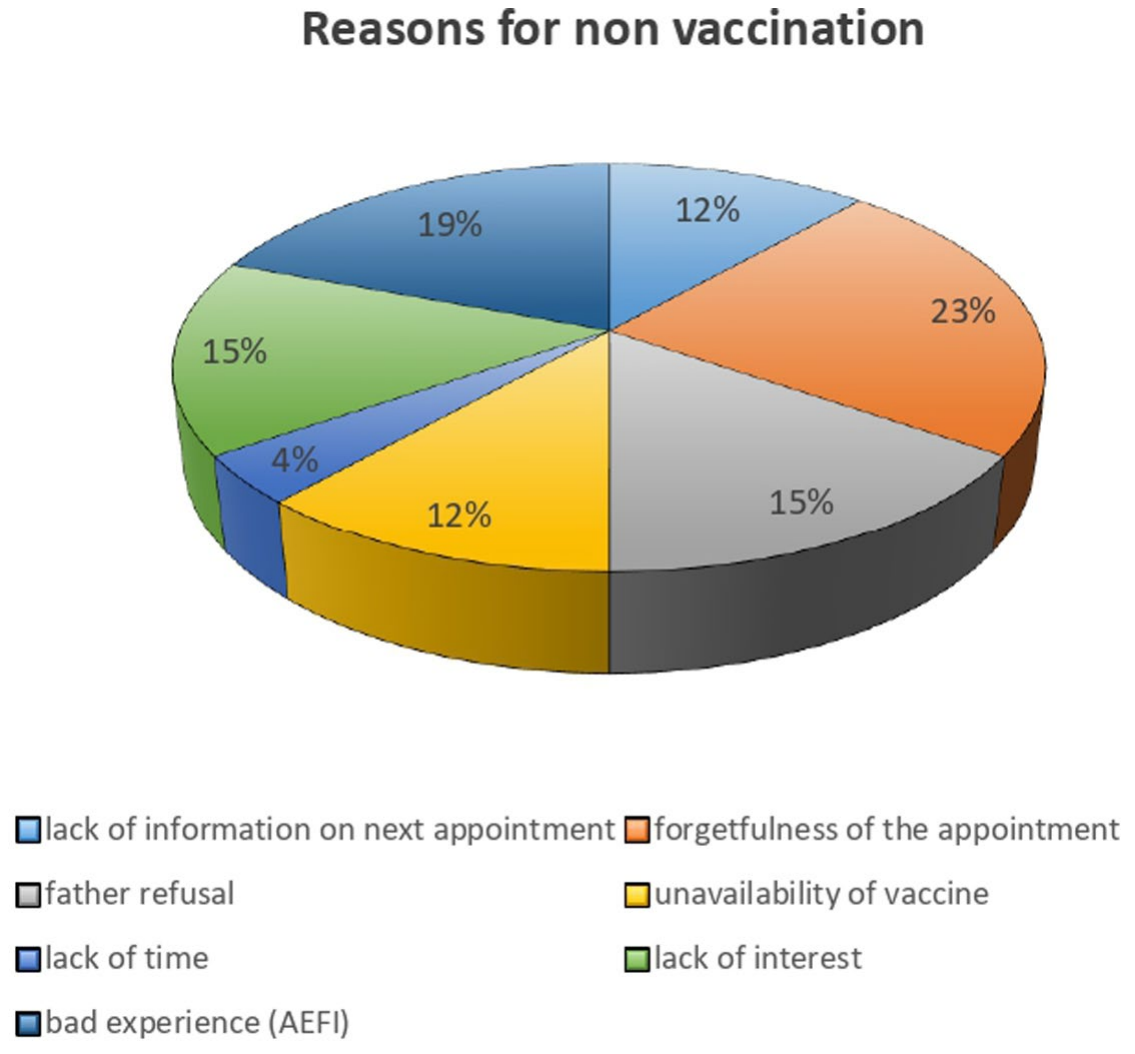
Reasons for non-vaccination (quantitative).

Several reasons for non-vaccination were reported by women with ZD/UI children within the studied health areas. Appointment forgetting was the most frequent reason (22%; *n* = 11), followed by adverse events following immunization (AEFI) (real or reported), especially in 18% (*n* = 9). Lack of interest in vaccination, husband opposition, and lack of time were reported in 16% (*n* = 8), 12% (*n* = 6), and 4% (*n* = 2) of cases, respectively. In addition to these personal reasons, participants also reported reasons related to health facilities: vaccine unavailability in 16% (*n* = 8) and lack of information about the next appointment in 12% (*n* = 6) (Figure 3).

Each mother could report several reasons.

### Qualitative analysis of non-vaccination causes

#### Causes related to vaccines and vaccination

**Figure 4:**
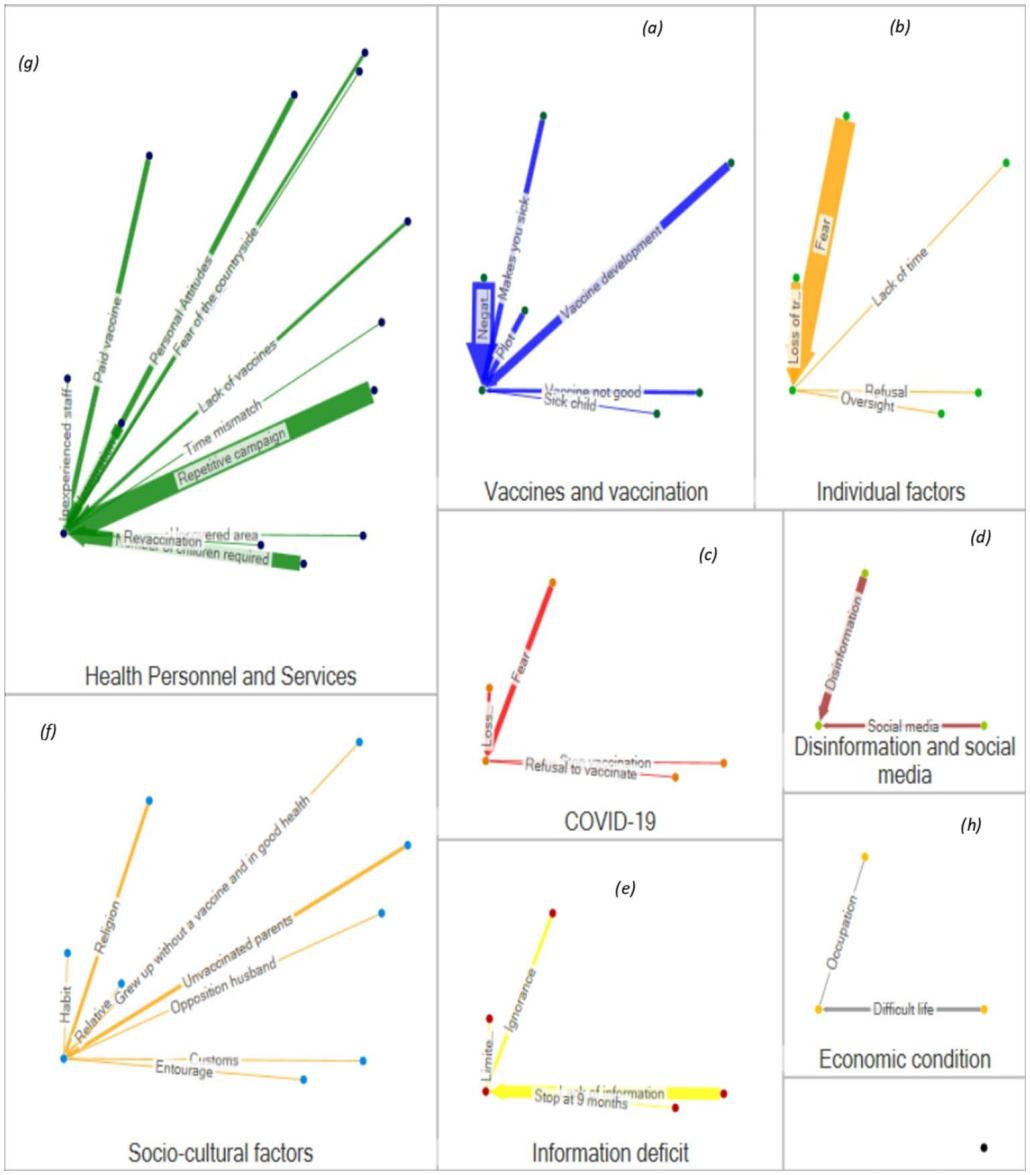
Reasons for non-vaccination and by group (qualitative). **(a)** Vaccines and vaccination; **(b)** Individual factors; **(c)** COVID‑19‑related factors; **(d)** Disinformation and social media; **(e)** Information deficit; **(f)** Socio‑cultural factors; **(g)** Health personnel and services; and **(h)** Economic conditions. Within each sub‑figure, edge widths correspond to the relative importance of factors as reported by participants.

This analysis revealed negative perceptions constituting important vaccination barriers. Personal negative experiences by mothers or their entourage were associated with mistrust toward vaccination, notably through apprehension of adverse effects. Some mothers questioned the quality of the vaccines administered, fearing they had expired or been contaminated. The analysis also highlighted fatigue from the repetition of vaccination campaigns, perceived by some as suspicious, which reinforced their doubts about the true motivations of these initiatives. One participant stated: “Each time we come, there are always problems with vaccines… we don’t know if they are safe anymore” (Figures 4a,b).

#### COVID-19 pandemic impact

The COVID-19 pandemic was associated with persistent disruptions in mothers’ vaccination behaviors. Temporary vaccination suspension during the pandemic was associated with prolonged disengagement for several families: “I had stopped having my daughter vaccinated when Covid was strong,*” as one mother’s testimony illustrates*.”

Fear of virus exposure in health facilities was identified as a factor associated with the interruption of vaccination follow-up beyond the acute health crisis, creating a rupture in preventive care continuity. Moreover, the perception that vaccination had become mandatory during the pandemic period provoked rejection among some mothers. As one participant explained, “During Covid, they told us we have to vaccinate, that it was mandatory. So, I said no…. that is my child” (Figure 4c).

#### Misinformation and social media influence

Analysis revealed the concerning role of social media in spreading false information about vaccination. Social media was reported as a channel that sometimes overrode official information from health authorities. Conspiracy theories, suggesting vaccination aimed at exterminating African populations, found resonance among some mothers. One participant’s testimony summarizes this problem: “The real problem is social media.” Another stated, *“I saw on WhatsApp that they put products in the vaccines to make our children sterile. It was shared by my people, I know*” R2.5 (Figure 4d).

#### Lack of information and inadequate sensitization

Results highlighted important gaps in information transmission regarding vaccination schedules and infant vaccination schedules. Unfamiliarity with the vaccination schedule appears as a recurring barrier, suggesting that mothers lack the basic knowledge necessary to ensure their children’s vaccination follow-up. Analysis also revealed that the existing sensitization strategies are inadequate for mothers’ real constraints, failing to account for their availability, specific concerns, or education levels. Moreover, information provided during prenatal and postnatal periods remains limited, thus missing a crucial health education opportunity when mothers are particularly receptive to messages concerning their child’s health (Figure 4e).

#### Emotional factors

The emotional dimension constitutes a major obstacle to vaccination, as the results demonstrate. Deep apprehension about vaccines, often disproportionate but nonetheless real for concerned mothers, paralyzes their capacity to take the vaccination step. This fear is accompanied by eroding confidence in the health system and the professionals who work there. Data also revealed more pragmatic but equally significant factors, such as forgetting vaccination appointments, which testifies to a lack of prioritization or organization. Finally, emotional fatigue with repetitive campaigns emerged from testimonies, suggesting that excessive solicitation can paradoxically lead to disengagement rather than adherence (Figure 4b).

#### Sociocultural factors

The analysis highlighted the importance of sociocultural determinants in the decision not to vaccinate. Religious convictions play a notable role; some mothers believe that prayer suffices to protect their children, thus rendering vaccination superfluous or even contrary to their faith. Individual perceptions reported during interviews do not reflect generalized religious positions. Family traditions also exert considerable influence, as illustrated by testimony stating, “Their parents grew up without vaccines; why vaccinate their children?” This logic, though epidemiologically erroneous, relies on previous generations’ lived experience and constitutes a persuasive argument in certain family contexts. Spousal opposition is another significant barrier, as mothers do not always have decision-making autonomy regarding their children’s health. Finally, weak integration of foreign communities into the local social and health fabric was associated with isolation, limiting their access to information and vaccination services (Figure 4f).

#### Health service and personnel dysfunctions

Results revealed multiple health service failures hindering vaccination. The health center’s opening hours appeared inadequate for mothers’ constraints, particularly for working mothers. The analysis also identified geographical areas not covered by vaccination services, creating access inequalities.

Frequent vaccine and consumable stock-outs compromised service continuity and eroded user confidence. Reception quality, judged deficient by several respondents, was an additional dissuasive factor, as was the unsanitary environment in some health facilities.

A particularly problematic practice emerged from the data: a minimum child threshold for opening a vaccine vial, which discouraged mothers presenting alone at the center. Finally, the illegal sale of vaccines that should be free constitutes a serious deviation undermining system confidence and creating a financial barrier. “Sometimes you come, and they tell you to wait for more children before they open the vial…. You end up going home without the vaccine,” said one participant (Figure 4g).

#### Personal and logistical constraints

Mothers face numerous personal constraints hindering their capacity to vaccinate their children. Lack of time appeared as a major barrier, particularly for working mothers. Transportation difficulties and long distances to reach health centers constituted logistical obstacles, particularly in urban areas where difficult-access zones exist, even by motorcycle in lowlands. “I work every day. I cannot lose my work to wait three hours at the health center,” explained one participant. Data also highlighted the competing priorities mothers face, notably the need to work to meet family needs or care for siblings, sometimes relegating vaccination to second place in the daily hierarchy of urgencies (Figure 4h).

### Obstacles encountered during identification and vaccination

Access to mothers constituted a major obstacle. In Essomba, 12 men came to “represent” their wives, preventing women’s direct participation. Overall, 18% of the families were represented by men. This pattern reflects structural constraints on women’s autonomous participation in health research and is consistent with documented gender barriers to maternal and child health services in sub-Saharan Africa. One-fifth of identified mothers (21%) refused to participate. Language barriers affected 4% of participants (three women spoke neither French nor English). Two women interrupted the interview, stating they needed their husbands’ prior authorization.

All Nkolo community organizations were initially male, as were half of those in Essomba and Mimboman 2. A second search achieved 75% women leaders in the three health areas. However, vulnerable communities consisting mainly of refugees (Central Africans) and internally displaced persons (from the NOSO crisis) were not represented in any health area.

Actor motivation varies between health areas. Essomba community health workers expected financial incentives; their involvement remained low. In Nkolo, involvement was high without financial incentives.

Geographic accessibility also poses problems. Nkolo and Mimboman 2 had landlocked zones identified at the health district level. None were visited or covered by the intervention. Poor road conditions prevent access, especially during the rainy season.

Time constraints reduced the intervention duration by 44%; 9 months were planned, but only 5 were completed. The before/after evaluation was contracted for 1 month (Table 2).

**Table 2:** Summary of barriers by study area.

Type of barriers	Nkolo	Essomba	Mimboman 2	Overall impact *n* (%)
Limited access to mothers				
Men attending “on behalf of” wives	0	12	0	12/68 (18%)
Refusal to participate				
Participants who declined	5	8	10	23/112 (21%)
Language barriers				
Women with no French/English proficiency	2	0	1	3/68 (4%)
Interview interrupted (husband’s permission required)	2	0	0	2/68 (3%)
Stakeholder motivation				
CHWs with financial expectations	No	Yes	No	1/3 sites
Active CHW engagement	High	Low	Moderate	Variable
Geographic accessibility				
Hard-to-reach areas identified	Yes	No	Yes	2/3 sites
Hard-to-reach areas covered	No	–	No	0/2 areas
Time constraints				
Planned intervention duration (months)	9	9	9	–
Actual duration achieved (months)	5	5	5	–44%

## Discussion

### Sociodemographic characteristics of mothers ZD/UI

The results showed that 23% of children in our sample were zero-dose. There was a predominance of single mothers (74%), a majority with a secondary education level (70%), and a high proportion of families living in poverty (66% with less than 50,000 FCFA monthly). These observations concur with several studies conducted in sub-Saharan Africa. Ozigbu et al. (2022) conducted multi-country analysis, covering 43,131 children from 33 sub-Saharan African countries, found that 16.5% had received no vaccines (11).

However, the profile of the mother of a ZD/UI child in this urban area differed slightly from what is typically known (11). A study covering 82 low- and middle-income countries found that 75.8% of children who received no doses lived in rural areas and 62.7% belonged to the two lowest wealth quintiles (12). In fact, this study population, despite being poor, had a secondary level of education and lived in an urban area. Most of them were single mothers with precarious jobs (informal sector) or no job. The finding of a high parental single status prevalence (74%) is particularly concerning, as it suggests a lack of family support and increased socioeconomic vulnerability. It also highlights the fact that, although important, women’s education alone is not enough. Financial autonomy plays a key role, especially in this urban area. Women’s precarity related to non-vaccination is multifactorial, as reported by Galadima et al. (13) and should be addressed globally.

### Reasons for non-vaccination

#### Appointment forgetting

Women reported appointment cancellations and lack of time as reasons for non-vaccination. This is consistent with the profile of an overwhelmed single mother lacking family support. Negative experiences (real or supposed) with AEFI were also reported as a barrier. These results echo Bangura et al.’s (2020) systematic review findings on vaccination barriers in sub-Saharan Africa, which emphasized that lack of awareness and information, as well as concerns about potential side effects, constitute major obstacles to vaccine acceptance (14). The importance of AEFI in this study reflects a major concern identified in several African contexts, where apprehension of adverse effects has been reported as one of the main vaccine hesitancy factors (15). These missed opportunities related to forgetting could be addressed during routine consultations. According to Olorunsaiye et al. (16), only 14−44% of care providers in four African countries (Kenya, Tanzania, Senegal, and Malawi) assessed children’s vaccination status, representing significant missed opportunities (16). To this, insufficient information on AEFI, misinformation about vaccines themselves was added.

The qualitative analysis showed that the COVID-19 pandemic had a significant negative impact on vaccination behaviors, leading to a temporary suspension that became prolonged disengagement for several families and exacerbating misinformation. Abbas et al. (17) conducted a benefit–risk analysis in Africa, showing that for each additional COVID-19 death, 84 child deaths could be prevented by maintaining routine childhood vaccination. Chelo et al. (18) reported in Cameroon in 2021 a significant decrease in demand for health services, associated with increased mortality among children during the COVID-19 pandemic. Three years after the pandemic’s start, the WHO African region had not yet recovered from COVID-19-caused disruptions, with 28.7 million zero-dose children recorded between 2019 and 2022 (19). Mothers also reported fear of immunization related to the COVID-19 pandemic.

#### Misinformation and social media influence

According to this study, social media proved to be a vector for spreading false information, with conspiracy theories suggesting vaccination aimed at exterminating African populations. These results are consistent with Nah et al.’s 2023 findings in Cameroon, which showed that social media use and medical mistrust were positively associated with belief in COVID-19 vaccine-related misinformation (20).

The advent of the pandemic led to an “infodemic,” characterized by widespread misinformation about the virus, its treatments, and vaccines, constituting a critical public health threat (20, 21). Behavioral interventions have been proposed, including “debunking” (fact-checking after exposure), “pre-bunking” (teaching about how fake news works before exposure), and accuracy prompts to encourage users to consider the veracity of content before sharing it (22). COVID-19-related fear should be considered in urban areas when providing immunization information.

### Sociocultural factors and vaccination

The data highlight the importance of religious beliefs, family traditions, and marital opposition in the decision not to vaccinate. Although reported in only a few cases in this study population, this is not negligible. A systematic review of childhood vaccination barriers in sub-Saharan Africa emphasized the importance of considering social contexts when designing and implementing childhood vaccination programs and of involving the entire community, not just mothers and female caregivers (13). Male partners’ attitudes about vaccination have been frequently noted, suggesting that decision-making power and women’s autonomy are relevant to maternal and child health outcomes. A study conducted in West Africa revealed that home birth, mothers’ lack of media access, absence of religion, poverty, and illiteracy were associated with incomplete child vaccination (23).

### Health services dysfunctions

The results of this study revealed multiple failures, including inadequate schedules, stock-outs, deficient reception quality, and an unsanitary environment. These findings are largely corroborated by Burnett et al.’s (24) hospital study in South Africa, which demonstrated that vaccine stock-outs constituted a major obstacle to missed opportunities for vaccination in children. The most common systemic failures included vaccine unavailability, stock-outs, and healthcare providers’ reluctance to open vials for fear of wastage. A study conducted in Kampala, Uganda, demonstrated that complex health system barriers to childhood vaccination persist even in urban settings, underscoring that hard-to-reach populations persist even in areas with good physical access.

### Personal and logistical constraints

Lack of time, transportation difficulties, and geographical inaccessibility (long distances) constituted major obstacles to our study. Despite being in an urban area, the studied health areas in the Nkolndongo Health District faced logistical difficulties and overcrowding, with impractical roads, especially during the rainy season. Bangura et al.’s 2020 systematic review reported that caregivers traveled long distances to reach vaccination centers, resulting in incomplete vaccination series (23). Vaccine hesitancy was higher when vaccination centers were difficult to access. Access and vaccination service experiences, including undesirable service characteristics, long waiting times, and financial implications, influenced vaccination decisions and practices in Africa.

### Study limitations and strengths

This study has several strengths, notably the use of a mixed-methods approach combining both quantitative and qualitative analyses, which allows a deep understanding of vaccination barriers. First, the small sample size (*n* = 50) limits the generalizability of the quantitative findings. Second, possible selection bias cannot be excluded. The most isolated or reluctant women, including those prevented by spouses, were systematically underrepresented. Third, refugee and internally displaced communities were entirely absent from the sample, represent a critical gap given their documented vulnerability to zero-dose status. Fourth, several landlocked geographical zones were not reached due to road conditions, potentially excluding the highest-risk populations. Fifth, recall bias is possible for reported AEFI experiences, and the use of maternal recall in the absence of vaccination cards introduces uncertainty regarding vaccination status classification. Some mothers were presented as “husband” or “grandmother,” reducing the sample size, as they were all excluded. The language barrier seems to be the reason for the withdrawal of three participants. These limitations reinforce the exploratory nature of this study and the need for larger and more representative studies.

## Conclusion

The profile of women with ZD/UI in Nkolndongo HD is that of single mothers with a secondary level of education living in precarity. Reasons for non-vaccination include misinformation, fear of vaccine safety and security, and health service dysfunctions. Addressing zero-dose children requires multifaceted interventions targeting misinformation, strengthening health systems, adapting services to mothers’ constraints, and engaging entire communities.

## Data Availability

The datasets presented in this article are not readily available because the data collected in this study is anonymous, as are the audio recordings. They are all kept secure. They will be used for publications and to develop strategies to improve vaccination coverage in our country. Requests to access the datasets should be directed to Ngo Um Sap, suzysap@gmail.com.
